# Deupirfenidone compared with pirfenidone and placebo in idiopathic pulmonary fibrosis (ELEVATE-IPF): a phase 2b randomized placebo-controlled trial

**DOI:** 10.1093/ajrccm/aamag155

**Published:** 2026-04-02

**Authors:** Toby M Maher, Mark J Hamblin, Won-Il Choi, Amy Hajari Case, Ioannis P Tomos, Argyrios E Tzouvelekis, Jessica E Shore, Miguel A Bergna, David Golod, Eric Elenko, Yanqiong Zhang, Camilla S Graham, Jin Woo Song, Tejaswini Kulkarni, and the ELEVATE IPF Investigators, Martin Maillo, Miguel Bergna, Ramon A Rojas, Luis Wehbe, Alexis Cazaux, Alejandro Chirino, Pedro Carlos Elias, Luciana Molinari, Alicia Molina, Cristian Fazio, Victoria Kohn, German Arce, Ernesto Raso, Matias Florenzano, Georgina Miranda, Juana Pavie Gallegos, Absalon Rafael Silva Orellana, Diana Cano, Tatiana Valencia Castano, David Tchkonia, Kakha Vacharadze, Vakhtang Katsarava, Nani Gonjilashvili, Lali Kupreishvili, Katerina Antoniou, Aikaterini Manika, Argyrios Tzouvelekis, Ioannis Tomos, Efrosyni Manali, Jaydip Deb, Sandeep Katiyar, Anjali R Nath, Hafiz Deshmukh, Tejas Kakkad, Rajesh Swarnakar, Gururaj Udachankar, Asish Deshmukh, Syazatul Syakirin Sirol Aflah, Yong-Kek Pang, Noorul Afidza Muhammad, Megat Razeem Abdul Razak, Irfhan Ali Hyder Ali, Chan Tha A Hing, Andrea Colli, Juan Francisco Moreno, Francisco Sanchez Llamas, Rodolfo Posadas Valay, Carlos Herrera Garcia, Joel Santiaguel, Mae Campomanes, Pamela Joy Dionisio, Cristian Oancea, Lavinia Davidescu, Cristian Cojocaru, Riaz Suleman Dawood, Ismail Kalla, Larry Mey, Michael Van Der Linden, Paul Graham Williams, Jin Woo Song, Yong Hyun Kim, Hyoung Kyu Yoon, Eun Kyung Kim, Hye Sook Choi, Sun Hyo Park, Hongseok Yoo, Won-Il Choi, Yongchul Lee, Yangjin Jegal, Sung Hwan Jeong, Hong-Joon Shin, DongWon Park, Jae Ha Lee, Joo Hun Park, Kittima Bangpattanasiri, Pailin Ratanawatkul, Krittika Teerapuncharoen, Thomas P Jensen, Barbara Wendelberger, Farah Khandwala, Anna McGlothlin, Aaron Milstone, Abhishek Singla, Juan Fernandez, Mark Hamblin, Tejaswini Kulkarni, Rafael Lupercio, Benjamin Bregman, Richard Parisi, Yolanda Mageto, Damien Patel, Toby Maher, William Stringer, Ather Siddiqi, Shilpa Johri, Gerard Criner, Bruce Rankin, Ramana Puppala, Amy Case, Ryan Klein, Murali Ramaswamy, Lisa Lancaster, Todd Astor, Ameer Rasheed

**Affiliations:** Imperial College, National Heart and Lung Institute, London, United Kingdom; Keck School of Medicine, University of Southern California, Los Angeles, CA, United States; Department of Internal Medicine, University of Kansas Medical Center, Kansas City, KS, United States; Department of Internal Medicine, Myongji Hospital, Hanyang University, Goyang-si, Republic of Korea; Department of Pulmonary, Critical Care, and Sleep, Piedmont Healthcare, Atlanta, GA, United States; 5th Pulmonary Medicine Department, SOTIRIA Chest Diseases Hospital of Athens, Athens, Greece; Department of Respiratory Medicine, University of Patras, Patras, Greece; Pulmonary Fibrosis Foundation, Chicago, IL, United States; Department of Research, CEMER, Florida, Vicente López, Argentina; PureTech Health, Boston, MA, United States; PureTech Health, Boston, MA, United States; PureTech Health, Boston, MA, United States; PureTech Health, Boston, MA, United States; Department of Pulmonary and Critical Care Medicine, Asan Medical Center, University of Ulsan College of Medicine, Seoul, Republic of Korea; Department of Medicine, University of Alabama at Birmingham, Birmingham, AL, United States

**Keywords:** interstitial lung disease, clinical trial, forced viral capacity, deuteration

## Abstract

**Rationale:**

Deupirfenidone is a strategically deuterated form of pirfenidone that retains pharmacodynamic activity but has a differentiated pharmacokinetic profile that may enable improved efficacy and favorable tolerability in patients with idiopathic pulmonary fibrosis (IPF).

**Objectives:**

To evaluate the efficacy and safety of deupirfenidone compared with placebo and pirfenidone in patients with IPF.

**Methods:**

Patients were randomized 1:1:1:1 to deupirfenidone 550 mg TID, deupirfenidone 825 mg TID, pirfenidone 801 mg TID, or placebo. The primary endpoint was the rate of change in forced vital capacity (FVC) for the combined arms of deupirfenidone versus placebo at 26 weeks. The primary and secondary analyses used Bayesian and frequentist approaches, respectively.

**Measurements and Main Results:**

A total of 257 patients with IPF were randomized, and the proportion on treatment at the end of the study was 80.0%, 68.3%, 64.6%, and 78.1% for the placebo, pirfenidone, deupirfenidone 550 mg, and deupirfenidone 825 mg arms, respectively. Posterior mean change in FVC for placebo was -110.71 mL (95% credible interval (CI), -148.75, -70.98), and for the combined deupirfenidone arms was -48.42 mL (95% CI, -87.66, -9.04) with a posterior mean difference of 62.29 mL (95% CI l, -6.13, 115.73; posterior probability, 0.985). Using a frequentist approach, the adjusted mean change in FVC for the placebo arm was -112.5 mL (95% CI, -167.2, -57.8), and for the deupirfenidone 825 mg arm was -21.5 mL (95% CI, -78.2, 35.1); the adjusted mean difference was 91.0 mL (95% CI, 12.2, 169.7; *P* = .02). The most common adverse events for each active treatment arm were gastrointestinal.

**Conclusions:**

In patients with IPF, treatment with deupirfenidone slowed lung disease progression over 26 weeks.

**Trial Registration:**

Clinicaltrials.gov number NCT05321420.

At a Glance Commentary **Current Scientific Knowledge on the Subject:** This randomized, double-blinded, active and placebo-controlled phase 2 trial assessed the effect of deupirfenidone, an oral, deuterated form of pirfenidone, on the rate of decline in forced vital capacity (FVC) over 26 weeks compared with placebo in patients with idiopathic pulmonary fibrosis (IPF). Compared with placebo, the deupirfenidone 825 mg dose reduced the rate of decline in FVC by 91 mL (p=0.02) and the safety profile was similar to that demonstrated in the pirfenidone arm.

**What This Study Adds to the Field:** These results support further evaluation of deupirfenidone as a treatment option for patients with IPF in a phase 3 trial.

## Introduction

Idiopathic pulmonary fibrosis (IPF) remains an invariably progressive and fatal disease despite the availability of pirfenidone and nintedanib in the United States since 2014. While both drugs have been shown to slow disease progression and improve transplant-free survival, neither halts disease worsening.^1-5^ Their limited efficacy, combined with tolerability challenges, contributes to low treatment uptake, frequent discontinuation of both treatments, and impaired quality of life.^6-8^ Improved treatment efficacy without compromising tolerability remains an unmet need for patients with IPF.

Pirfenidone is a small molecule that modulates fibrotic and inflammatory pathways, including TGF-β and TNF-α.^9^^,^^10^ The efficacy of pirfenidone is dose-dependent but adverse effects are often dose-limiting and largely related to gastrointestinal events.^2^ Deupirfenidone is a selectively deuterated analog of pirfenidone, with three deuterium (heavy hydrogen) atoms replacing hydrogen in the methyl group.^11^ Deupirfenidone has been shown to have the same pharmacodynamic effects as pirfenidone but with a differentiated pharmacokinetic profile.

We conducted phase 2 b ELEVATE IPF trial to investigate the safety, tolerability, and efficacy of two doses of deupirfenidone (550 mg and 825 mg), both three times daily (TID), compared to placebo and an active control arm consisting of pirfenidone 801 mg TID in patients with IPF.

## Methods

### Trial design and oversight

ELEVATE IPF was a 4-arm, randomized, double-blind, active- and placebo-controlled, dose-finding, phase 2 b trial that enrolled patients from 87 sites in 14 countries (Figure E1; Online Data Supplement list of ELEVATE IPF investigators). Trial conduct was compliant with the protocol and the Declaration of Helsinki and the ICH Integrated Addendum to E6 (R1): Guideline for Good Clinical Practice ICH E6 (R2). The study was approved by Institutional Review Boards (IRB) for each site. Patients provided written informed consent before trial entry. The trial was designed by the Clinical Advisory Committee (listed in the Supplement) and sponsor, PureTech Health.

### Patients

The first patient was screened on August 22, 2022 and the last patient completed the week 26 visit on October 15, 2024. Eligible patients were aged ≥ 40 years, antifibrotic treatment-naive or had prior receipt of nintedanib for no more than 6 months and were no longer on antifibrotic treatment and had physician-diagnosed IPF based on current international criteria with centrally adjudicated definite or probable usual interstitial pneumonia pattern on a CT scan performed within 12 months of screening. Patients had to have an FVC ≥ 45% of predicted value, and, if available at the study site, diffusing capacity of the lungs for carbon monoxide (DLCO) corrected for hemoglobin (Hb) ≥ 30% and ≤ 90% of predicted. Detailed inclusion and exclusion criteria are provided in the Supplement Methods.

### Trial protocol

Randomization was performed with the use of an interactive web-response system with permuted blocks and stratified based on prior exposure to nintedanib or nintedanib-naive status. Patients were randomly assigned 1:1:1:1 to receive either deupirfenidone 550 mg TID, deupirfenidone 825 mg TID, pirfenidone 801 mg TID, or matching placebo. The administered dose for all four arms was titrated up to the target dose at pre-specified intervals over three weeks. Subsequently, dose adjustments were allowed in response to safety findings or tolerability issues. After the 26-week blinded period, eligible patients were offered enrollment into an open-label extension trial; those who declined underwent a final visit 4 weeks after the last dose of the investigational drug. Pulmonary function was assessed at screening, baseline, and at weeks 4, 8, 16, and 26. Physical examination, clinical laboratory assessments, and safety and efficacy assessments, including adverse event monitoring and vital signs, were conducted throughout the study at regular intervals. An Independent Data Monitoring Committee (members listed in Supplement) reviewed unblinded data after 40, 100, and 200 patients completed 4 weeks on treatment.

### End points

The primary endpoint was the rate of decline in FVC (in mL) over 26 weeks. The key secondary efficacy endpoint was the rate of decline in FVC percent of predicted (FVCpp) over 26 weeks. Other secondary endpoints are detailed in the Supplement and included time to IPF progression through 26 weeks as defined by an absolute decline from baseline in FVCpp of 5% or greater or death, incidence of dose modifications (dose reductions or interruptions), and incidence of adverse events of special interest (AESI).

The safety dataset included all patients who received at least one dose of study drug. Safety was assessed through recording of adverse events and clinical monitoring. The frequency and severity of adverse events were documented according to the Medical Dictionary for Regulatory Activities, version 25.0 and were graded using the Modified National Cancer Institute Common Terminology Criteria for Adverse Events (mCTCAE).

### Statistical analyses

Efficacy analyses were assessed using the Full Analysis Set (FAS), which was defined as all randomized participants who received at least one dose of study drug and had at least one valid efficacy assessment after baseline. For spirometry assessments, only grade A and B FVC measurements, as determined by central read/adjudication, were considered valid for analysis.^12^

The primary efficacy analysis was performed using a Bayesian linear mixed effects model. The response variable was absolute measurements over time, including baseline. Fixed effects included treatment, time in weeks (as a continuous variable), and treatment-by-time interaction, while random effects were included for subject-level intercepts and slopes. The Bayesian approach incorporated dynamic borrowing of historical placebo data from several external IPF trials, including the ASCEND, INPULSIS, and TOMORROW studies (Figures E2 and E3).^2^^,^^3^^,^^13^ These trials were selected based on comparable placebo population characteristics, inclusion/exclusion criteria, and background therapy profiles. Prior specifications for dynamic borrowing are described in the Supplement and Table E1. The primary analysis evaluated the superiority of the pooled deupirfenidone arms compared with the placebo arm.

Pre-specified secondary analyses of the primary endpoint data used a linear mixed effects model with the same structure as the Bayesian model. Missing data were assumed to be missing at random (MAR). No imputation was performed; analyses were conducted using available data only. For longitudinal outcomes, mixed-effects models provide valid estimates under the MAR assumption. No penalty was imposed for death. Both absolute FVC (mL) and FVCpp were analyzed using Bayesian and frequentist approaches to provide complementary insights.

Time-to-event endpoints were analyzed using Kaplan-Meier methodology with log-rank tests and Cox regression performed with the modified GAP index Global as the explanatory variable. Further information on the statistical analyses is provided in the Supplement. The sample size calculation is described in the Supplement.

## Results

### Patients

**Figure 1:**
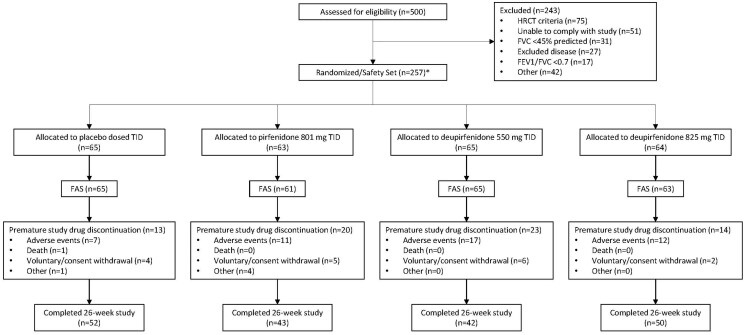
Study design. HRCT = High resolution computed tomography; FVC = Forced vital capacity; FEV1 = Forced expiratory volume during the first second; TID—three times daily; FAS = Full analysis set (all randomized participants who received at least one dose of study drug and had at least one valid efficacy assessment after baseline)

A total of 257 patients were randomized to one of the four investigational arms and received at least one dose of trial drug (Figure 1). Baseline characteristics were similar across the 4 treatment groups (Table 1). Mean (±SD) age was 70.9 (7.98) years with a mean FVCpp of 78.94 (19.61). The trial population generally reflected the characteristics of patients with IPF (Table E2). A total of nine patients had previously taken nintedanib therapy. The percentage of FVC tests that were Grade A or B (used for efficacy analyses) were > 88% for all arms.

**Table 1:** Characteristics of the randomized patients at baseline according to treatment arm.

Characteristic	**Placebo TID (N** = **65)**	**Pirfenidone 801 mg TID (N** = **63)**	**Deupirfenidone 550 mg TID (N** = **65)**	**Deupirfenidone 825 mg TID (N** = **64)**	**Overall (N** = **257)**
**Age, mean, (SD)**	71.7 (7.27)	71.0 (8.50)	70.9 (7.89)	70.0 (8.31)	70.9 (7.98)
**Age group, n (%)**					
**<65 years**	8 (12.3)	14 (22.2)	13 (20.0)	17 (26.6)	52 (20.2)
**≥65 and < 75**	36 (55.4)	22 (34.9)	31 (47.7)	25 (39.1)	114 (44.4)
**≥75**	21 (32.3)	27 (42.9)	21 (32.3)	22 (34.4)	91 (35.4)
**Male**	47 (72.3)	47 (74.6)	46 (70.8)	43 (67.2)	183 (71.2)
**Female**	18 (27.7)	16 (25.4)	19 (29.2)	21 (32.8)	74 (28.8)
**Race^a^, n (%)**					
**White or Caucasian**	38 (58.5)	42 (66.7)	40 (61.5)	42 (65.6)	162 (63.0)
**Asian**	22 (33.8)	21 (33.3)	22 (33.8)	21 (32.8)	86 (33.5)
**Black or African American**	3 (4.6)	0	1 (1.5)	0	4 (1.6)
**Other**	2 (3.1)	0	2 (3.1)	1 (1.6)	5 (1.9)
**Ethnicity, n (%)**					
**Hispanic or Latino**	23 (35.4)	14 (22.2)	16 (24.6)	14 (21.9)	67 (26.1)
**Region, n (%)**					
**United States**	10 (15.4)	20 (31.7)	13 (20.0)	11 (17.2)	54 (21.0)
**Central/South America**	23 (35.4)	16 (25.4)	17 (26.2)	14 (21.9)	70 (27.2)
**Europe and South Africa**	10 (15.4)	8 (12.7)	14 (21.5)	19 (29.7)	51 (19.8)
**Asia**	22 (33.8)	19 (30.2)	21 (32.3)	20 (31.3)	82 (31.9)
**BMI^b^ (kg/m2; mean, SD)**	27.39 (5.079)	27.60 (4.018)	26.96 (4.788)	27.12 (4.942)	27.26 (4.708)
**Prior nintedanib use < 6 months, n (%)**	1 (1.5)	2 (3.2)	3 (4.6)	3 (4.7)	9 (3.5)
**Years of IPF diagnosis (mean, SD)**	1.4 (1.75)	1.8 (2.50)	1.8 (2.42)	2.1 (2.30)	1.8 (2.26)
**IPF diagnosis < 2 years, n (%)**	51 (78.5)	45 (71.4)	46 (70.8)	39 (60.9)	181 (70.4)
**HRCT pattern, n (%)**					
**Probable UIP**	31 (47.7)	32 (50.8)	31 (47.7)	32 (50.0)	126 (49.0)
**UIP**	34 (52.3)	31 (49.2)	34 (52.3)	32 (50.0)	131 (51.0)
**Baseline FVC (mL) mean, SD**	2550.5 (974.01)	2682.6 (729.79) (n = 61)	2672.9 (845.35)	2659.2 (871.70) (n = 63)	2640.5 (857.99)
**Baseline FVCpp, mean, SD**	76.74 (19.822)	79.52 (17.203) (n = 61)	80.11 (20.438)	79.45 (20.951) (n = 63)	78.94 (19.610)
**Baseline FVC pp < 50%, n (%)**	3 (4.6)	3 (4.9)	1 (1.5)	5 (7.9)	12 (4.7)

TID = three times daily; SD = standard deviation; IPF = Idiopathic pulmonary fibrosis; HRCT = High-resolution computed tomography; UIP = Usual interstitial pneumonia; FVC = Forced vital capacity; FVCpp = percent of predicted value.

a Race was recorded in the electronic case-report form by the trial site staff without instructions for the determination of race.

b BMI = Body-mass index; weight in kilograms divided by the square of the height in meters.

Similar proportions of patients in each treatment group continued treatment until week 26; 52 (80.0%) on placebo, 43 (68.3%) receiving pirfenidone 801 mg, 42 (64.6%) receiving deupirfenidone 550 mg, and 50 (78.1%) receiving deupirfenidone 825 mg (Figure 1).

### Efficacy

#### Pooled deupirfenidone arms compared with placebo

**Figure 2:**
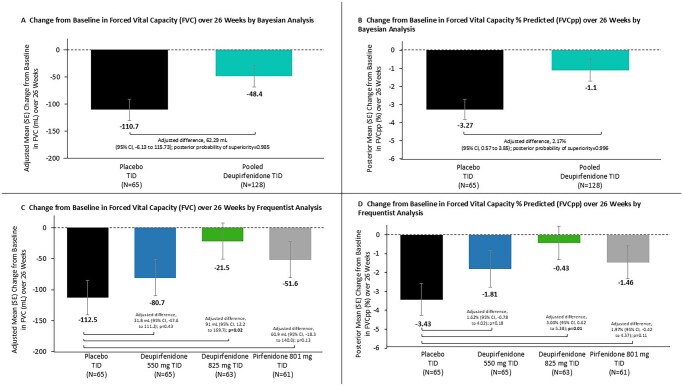
Changes from baseline over 26 weeks for A. FVC by bayesian analysis; B. FVCpp by bayesian analysis; C. FVC by frequentist analysis; and D. FVCpp by frequentist analysis. FVC = forced vital capacity; FVCpp = percent of predicted value; SE = standard error; TID = 3 times daily. Posterior mean (SE) by Bayesian approach is estimated based on a random coefficient regression model with absolute FVC (A) or FVCpp (B) over time, including baseline, as a response, and fixed effects for treatment (placebo or pooled deupirfendione 550 mg and deupirfenidone 825 mg), week, and treatment by week interaction, as well as participant-level random effects for the intercept and slope. Adjusted mean (SE) by frequentist approach is estimated based on a random coefficient regression model with absolute FVC (C) or FVCpp (D) over time, including baseline, as a response, and fixed effects for treatment (placebo, pirfenidone, deupirfenidone mg or deupirfenidone 825 mg), week, and treatment by week interaction, as well as participant-level random effects for the intercept and slope.

The posterior mean change in absolute FVC from baseline to week 26 using Bayesian analysis for placebo was -110.71 mL (95% credible interval, -148.75, -70.98), and for the pooled deupirfenidone arms was -48.42 mL (95% credible interval, -87.66, -9.04) (Figure 2A). When compared to the placebo arm, the posterior mean difference for the pooled deupirfenidone arms was 62.29 mL (95% credible interval, -6.13, 115.73; posterior probability of superiority, 0.985). A similar high posterior probability of superiority (99.6%) was demonstrated for the posterior mean change in FVCpp for the pooled deupirfenidone arms compared with placebo (Figure 2B).

#### Deupirfenidone 825 mg TID compared with placebo

Using a frequentist approach, the adjusted mean change in FVC for the placebo arm was -112.5 mL (95% CI, -167.2, -57.8) and for the deupirfenidone 825 mg arm was -21.5 mL (95% CI, -78.2, 35.1). When compared to the placebo arm, the adjusted mean difference for the deupirfenidone 825 mg arm was 91.0 mL (95% CI, 12.2, 169.7; *P* = .02) (Figure 2C, Figure E4, and Table E3). A similar analysis using rate of change of FVCpp demonstrated an adjusted mean change for the placebo arm of -3.43 (95% CI -5.08, -1.77) and for deupirfenidone 825 mg TID of -0.43 (95% CI -2.14, 1.29). When compared to the placebo arm, the adjusted mean difference for FVCpp for the deupirfenidone 825 mg arm was 3.00 (95% CI 0.62, 5.38; *P* = .01) (Figure 2D, Figure E5, and Table E4).

Time to IPF progression, defined as greater than 5% decline in FVCpp over 26 weeks or death, was significantly delayed for the deupirfenidone 825 mg treated arm (HR 0.439, 95% CI, 0.255, 0.756, *P* = .0023) compared to placebo (Figure E6).

#### Deupirfenidone 825 mg TID compared with deupirfenidone 550 mg TID

Using a frequentist approach, deupirfenidone 825 mg TID had an adjusted mean change in absolute FVC of -21.5 mL (95% CI, -78.2, 35.1) compared with deupirfenidone 550 mg arm of -80.7 mL (95% CI, -138.3, -23.1), demonstrating a dose response. Similarly, deupirfenidone 825 mg TID had an adjusted mean change in FVCpp of -0.43 (95% CI -2.14, 1.29) compared with deupirfenidone 550 mg arm of -1.81 (95% CI, -3.55, -0.07). Neither of these comparisons were statistically significantly different (Figure 2C and D).

#### Active control arm supported internal validity

Given differences in baseline characteristics between IPF studies, ELEVATE-IPF used not only a placebo control but also an active control, pirfenidone, to support internal study validity. The adjusted mean change in FVC for the pirfenidone arm was -51.6 mL (95% CI, -108.8, 5.6) and for the placebo arm was -112.5 mL (95% CI, -167.2, -57.8). When compared to the placebo arm, the adjusted mean difference for the pirfenidone 801 mg TID arm was 60.9 mL (95% CI, -18.3, 140.0; *P* = .13) (Figure 2C). An analysis using the rate of change of FVCpp demonstrated an adjusted mean change for the placebo arm of -3.43 (95% CI -5.08, -1.77) and for pirfenidone of -1.46 (95% CI -3.19, 0.28). When compared to the placebo arm, the adjusted mean difference for the pirfenidone 801 mg arm was 1.97 (95% CI -0.42, 4.37; *P* = .11, relative improvement compared with placebo of 57%) (Figure 2D).

### Safety

Adverse events during the treatment period from the first dose through 28 days after the last dose of the study drug are summarized in Table 2 (Table E5 describes preferred term groupings). Compared with placebo, gastrointestinal, neurologic, and cutaneous events were more common in the pirfenidone 801 mg and deupirfenidone 825 mg treatment arms. The incidence of these adverse events in the deupirfenidone 550 mg arm was intermediate between placebo and the other two treatment arms. Events were generally mild to moderate in severity, were reversible, and without clinically significant sequelae.

**Table 2:** Adverse event during treatment, including the 28-day follow-up period, according to treatment arm.

Adverse event^a^	**Placebo TID (N** = **65) n (%)**	**Pirfenidone 801 mg TID (N** = **63) n (%)**	**Deupirfenidone 550 mg TID (N** = **65) n (%)**	**Deupirfenidone 825 mg TID (N** = **64) n (%)**
**>=1 Treatment-emergent adverse event**	48 (73.8)	53 (84.1)	47 (72.3)	55 (85.9)
**All treatment-emergent serious adverse events**	10 (15.4)	6 (9.5)	12 (18.5)	7 (10.9)
**Treatment-emergent serious adverse events considered by investigator to be related to study drug**	2 (3.1)	1 (1.6)	0 (0)	1 (1.6)
**Prespecified adverse events of special interest^b^**	1 (1.5)	5 (7.9)	2 (3.1)	4 (6.3)
**Treatment-emergent adverse events leading to study treatment discontinuation**	8 (12.3)	11 (17.5)	16 (24.6)	12 (18.8)
**Treatment-emergent adverse events leading to any dose modifications^c^**	20 (30.8)	24 (38.1)	27 (41.5)	30 (46.9)
**Treatment-emergent adverse events leading to death**	2 (3.1)	5 (7.9)	1 (1.5)	1 (1.6)
**All-cause mortality^d^**	3 (4.6)	5 (7.9)	2 (3.1)	1 (1.6)
**SOC/PT^e^**				
**Gastrointestinal disorders**	16 (24.6)	33 (52.4)	23 (35.4)	34 (53.1)
**Nausea**	5 (7.7)	17 (27.0)	11 (16.9)	13 (20.3)
**Dyspepsia^f^**	2 (3.1)	14 (22.2)	8 (12.3)	9 (14.1)
**Diarrhea**	6 (9.2)	7 (11.1)	7 (10.8)	5 (7.8)
**Abdominal pain^f^**	3 (4.6)	5 (7.9)	4 (6.2)	9 (14.1)
**Constipation**	1 (1.5)	4 (6.3)	1 (1.5)	3 (4.7)
**Vomiting**	0 (0)	2 (3.2)	5 (7.7)	1 (1.6)
**Nervous system disorders**	7 (10.8)	11 (17.5)	12 (18.5)	13 (20.3)
**Dizziness**	2 (3.1)	5 (7.9)	6 (9.2)	8 (12.5)
**Headache**	3 (4.6)	8 (12.7)	5 (7.7)	2 (3.1)
**Skin disorders**	3 (4.6)	18 (28.6)	12 (18.5)	20 (31.3)

**Photosensitivity reaction**	0 (0)	5 (7.9)	4 (6.2)	5 (7.8)
**Rash^f^**	1 (1.5)	6 (9.5)	3 (4.6)	6 (9.4)
**Pruritus**	0 (0)	3 (4.8)	5 (7.7)	5 (7.8)
**Metabolism and nutrition disorders**	9 (13.8)	12 (19.0)	14 (21.5)	17 (26.6)
**Decreased appetite^f^**	5 (7.7)	9 (14.3)	12 (18.5)	13 (20.3)
**General disorders**	7 (10.8)	11 (17.5)	10 (15.4)	11 (17.2)
**Fatigue**	1 (1.5)	7 (11.1)	5 (7.7)	6 (9.4)
**Respiratory disorders**	23 (35.4)	12 (19.0)	13 (20.0)	15 (23.4)
**Cough**	7 (10.8)	3 (4.8)	1 (1.5)	8 (12.5)
**IPF**	10 (15.4)	2 (3.2)	3 (4.6)	4 (6.3)
**Dyspnea**	4 (6.2)	3 (4.8)	2 (3.1)	1 (1.6)
**Infections**	20 (30.8)	17 (27.0)	17 (26.2)	14 (21.9)
**Upper respiratory infections^f^**	6 (9.2)	9 (14.3)	8 (12.3)	6 (9.4)
**Urinary tract infections**	2 (3.1)	5 (7.9)	4 (6.2)	3 (4.7)
**Pneumonia**	3 (4.6)	2 (3.2)	4 (6.2)	1 (1.6)
**Psychiatric disorders**	1 (1.5)	5 (7.9)	3 (4.6)	7 (10.9)
**Insomnia**	0 (0)	3 (4.8)	1 (1.5)	4 (6.3)
**Highest ALT or AST increase**	N = 62	N = 62	N = 59	N = 61
**3 - ≤5 x ULN^g^**	1	1	1	1
**>5 - ≤8 x ULN**	1	0	1	0
**>8 ULN**	0	0	0	2

TID = three times daily.

a Adverse events were assessed with the use of the *Medical Dictionary for Regulatory Activities*, version 25.0.

b Adverse events of special interest included anorexia, decreased appetite, fatigue, diarrhea, nausea, vomiting, increase in aspartate aminotransferase (AST) and/or alanine aminotransferase (ALT) levels, photosensitivity reaction, or rash of grade 3 severity or higher.

c Dose modifications included temporary or permanent dose reductions, dose interruptions, and permanent study drug discontinuations.

d All-cause mortality included deaths that occurred more than 28 days after the last dose of study drug, so not classified as treatment-emergent.

e All System Organ Class (SOC) and Preferred Term (PT) where PT incidence of more than 5% in at least one treatment arm is reported.

f Dyspepsia, abdominal pain, rash, decreased appetite, and upper respiratory infections were grouped from multiple PTs. Please refer to the Preferred Terms Grouping Categories list in Table E5).

g ULN = Upper limit of normal, based on relevant local or central laboratory normal ranges for test values.

Gastrointestinal adverse events were the most common treatment-emergent adverse events (TEAE) reported in 16 patients (24.6%) in the placebo arm, 33 patients (52.4%) in the pirfenidone arm, 23 patients (35.4%) in the deupirfenidone 550 mg arm, and 34 patients (53.1%) in the deupirfenidone 825 mg arm, and the most common TEAE, nausea, occurred in 5 (7.7%), 17 (27.0%), 11 (16.9%) and 13 (20.3%) of patients, respectively. Liver enzyme abnormalities > 5 x ULN (the threshold for permanent study drug discontinuation) were reported in 1 patient on placebo, 1 on deupirfenidone 550 mg and 2 on deupirfenidone 825 mg arms. The cutaneous TEAEs of photosensitivity and rash were similar for pirfenidone and deupirfenidone 825 mg. Photosensitivity was experienced by 5 patients each on pirfenidone (7.9%) and deupirfenidone 825 mg (7.8%). Cough, dyspnea, and worsening of IPF generally occurred more frequently with placebo.

Grade 3 or higher TEAEs were uncommon (Table E6). Nine patients (13.8%) experienced at least one grade 3 or higher TEAE on placebo, 10 patients (15.9%) on pirfenidone, 13 patients (20.0%) on deupirfenidone 550 mg, and 8 patients (12.5%) on deupirfenidone 825 mg arms. Adverse events of special interest are listed in Table E7. The number (%) of patients with at least 1 treatment-emergent serious AE that was judged related to the study drug was 2 (3.1%) for placebo (cholangitis, gastroenteritis), 1 (1.6%) for pirfenidone (vomiting and decreased oxygen saturation), zero for deupirfenidone 550 mg, and 1 (1.6%) for deupirfenidone 825 mg (nausea), respectively (Table E8). All-cause mortality during the treatment period was lower in both deupirfenidone arms (1 patient (1.6%) in the 825 mg arm and 1 patient (1.6%) in the 550 mg arm) compared to pirfenidone (5 patients; 7.9%) and placebo (2 patients; 3.2%). No deaths were considered by investigators to be related to study drug. Table 2 provides the number of deaths during the treatment period and those that occurred more than 28 days after the last dose of study drug. Table E9 provides additional details about the deaths that occurred during the treatment period.

TEAEs that led to any dose modifications (temporary and permanent dose reductions, temporary dose interruptions, and permanent study drug discontinuations) occurred in 20 patients (30.8%) on placebo, 24 patients (38.1%) on pirfenidone 801 mg, 27 patients (41.5%) on deupirfenidone 550 mg, and 30 patients (46.9%) on deupirfenidone 825 mg. TEAES that led to treatment discontinuation occurred in 8 patients (12.3%) on placebo, 11 patients (17.5%) on pirfenidone 801 mg, 16 patients (24.6%) on deupirfenidone 550 mg, and 12 patients (18.8%) on deupirfenidone 825 mg (Table E10). The most common TEAEs leading to discontinuation (more than one patient by preferred term) for each treatment arm were IPF exacerbation for placebo, nausea, fatigue, headache, and pneumonia for pirfenidone, nausea, diarrhea, dyspepsia, fatigue, asthenia, IPF, and decreased appetite for deupirfenidone 550 mg, and nausea for deupirfenidone 825 mg arms, respectively.

## Discussion

ELEVATE-IPF demonstrated that deupirfenidone significantly slows FVC decline at 26 weeks compared with placebo. The active comparator arm, pirfenidone 801 mg TID, also slowed FVC decline compared to placebo with a relative effect size of 54% over placebo that was comparable to the effect size seen in the pivotal ASCEND trial, providing internal validity. Importantly, deupirfenidone 825 mg TID demonstrated a numerically superior benefit over both deupirfenidone 550 mg TID and pirfenidone 801 mg TID. The safety and tolerability profile of deupirfenidone was reassuring and consistent with that seen with pirfenidone.

Other clinical trials of investigational drugs in IPF have allowed background antifibrotic therapy, which were not allowed in this study.^15^^,^^16^ Since pirfenidone was included as one of the treatment arms, and deupirfenidone has similar pharmacodynamic properties as pirfenidone, pirfenidone could not be allowed as a background antifibrotic. Background nintedanib was not allowed because previous small clinical trials assessing the combination of nintedanib and pirfenidone have shown high rates of adverse events and discontinuation that could have compromised study integrity.^17^^,^^18^

Targeted deuteration has the potential to advantageously alter the pharmacokinetic properties of a drug. Deutetrabenazine, a deuterated form of tetrabenazine, was the first deuterated drug to be approved by the FDA in 2017, and since then, 3 other deuterated drugs have been approved in the United States.^19^ Deupirfenidone 550 mg TID results in plasma exposure that is relatively lower than pirfenidone 801 mg TID, while deupirfenidone 825 mg TID is a 50% higher dose that was included to test the impact of higher exposure on efficacy and tolerability. Of the three active treatment arms, deupirfenidone 825 mg TID demonstrated the largest treatment effect compared with placebo, slowing FVC decline over 26 weeks to a level approaching the normal physiological loss of FVC with aging.^14^ Since the approval of pirfenidone and nintedanib in 2014, trials conducted in patients with IPF have continued to compare active treatment to placebo, and thus, a direct comparison of efficacy between antifibrotic therapies has not been possible. ELEVATE IPF was not sufficiently powered to detect statistically significant treatment differences between individual treatment arms, nonetheless, the numerical difference in the adjusted mean difference in FVC for deupirfenidone 825 mg TID compared with pirfenidone is extremely encouraging and suggests that the increased plasma exposure achieved with deupirfenidone 825 mg TID is associated with improved efficacy.

Pirfenidone is known to be associated with gastrointestinal, neurologic, and cutaneous effects. As expected, adverse events in these 3 organ systems were higher in the 3 active treatment arms compared to placebo. The overall incidence of adverse events for deupirfenidone 825 mg TID was similar to pirfenidone 801 mg TID. The reported adverse events were generally mild to moderate in severity, reversible, and without clinically significant sequelae. The percentage of patients who remained on deupirfenidone 825 mg TID for 26 weeks (78.1%) was similar to the percentage of patients remaining on placebo (80.0%). These data suggest that the higher exposure and improved efficacy seen with deupirfenidone 825 mg TID was achieved without sacrificing tolerability.

The study has several limitations. Overall sample size and study duration, while powered to determine the impact of deupirfenidone on FVC, is inadequate to understand the true impact of deupirfenidone on secondary endpoints including hospitalizations, survival, and quality of life measures. The study was not powered to demonstrate the superiority of deupirfenidone over pirfenidone. Patients previously exposed to any dose of pirfenidone were excluded from participation in the study, and background nintedanib was not permitted. Although the number of deaths observed during the study was similar to previous trials, half of the deaths occurred in the pirfenidone arm. The resultant missing 26-week data may have impacted analyses, especially for the pirfenidone arm.

These results need to be confirmed in an adequately powered phase 3 study. Given the clear dose response seen between the deupirfenidone 550 mg and 825 mg doses, the 825 mg dose will be studied in phase 3. Dose modification will be allowed to address safety and tolerability issues, as was done in this study. A head-to-head superiority trial of deupirfenidone 825 mg TID and pirfenidone 801 mg TID will provide the most robust set of efficacy and safety data to allow patients living with IPF and clinicians to understand the potential value of deupirfenidone.

In conclusion, in this active- and placebo-controlled, dose-finding, phase 2 b trial, treatment with deupirfenidone 825 mg TID preserved lung function and demonstrated a favorable safety profile in patients living with IPF.

## Supplementary Material

aamag155_Supplementary_Data

## Data Availability

This article has an online data supplement, which is accessible from this issue’s table of content online at www.atsjournals.org.
