# Supplementary material for: Deupirfenidone compared with pirfenidone and placebo in idiopathic pulmonary fibrosis (ELEVATE-IPF): a phase 2b randomized placebo-controlled trial
Source: Am J Respir Crit Care Med. 2026 Apr 2;212(8):1761–9. doi: 10.1093/ajrccm/aamag155 (PMC13424664; doi:10.1093/ajrccm/aamag155)
Supplement: aamag155_Supplementary_Data [file aamag155_supplementary_data.zip › ELEVATE Online Data Supplement Blue J_FINAL_revisions_CLEAN.docx]

**Online Data Supplement**

**Deupirfenidone Compared with Pirfenidone and Placebo in Idiopathic Pulmonary Fibrosis (ELEVATE-IPF): a Phase 2b Randomized Placebo-Controlled Trial**

**Table of Contents** **Page**

**1. Investigators and Committee Members**

Members of the ELEVATE IPF Investigator Authorship Group ………………………………………………………............3

Clinical Advisory Committee Members…………………………………………………………………………………………………….9

Independent Data Monitoring Committee Members……………………………………………………………………………….9

**2. Supplementary Methods**

Key exclusion criteria……………………………………………………………………………………………………………………………….9

Further endpoints………………………………………………………………………………………………………………………………….10

Clinical trial sources and modeling used for Bayesian analyses……………………………………………………………...12

Sample size determination…………………………………………………………………………………………………………….........13

Data Source…………………………………………………………………………………………………………………………………………..14

**3. Supplementary Figures**

**Figure E1.** Study Design…………………………………………………………………………………………………………………………16

**Figure E2.** Estimates of Annual Rate of Change in FVC Volume (mL/week) from

Historical Placebo Data …………………………………………………………………………………………………………………………17

**Figure E3**: Estimates of Annual Rate of Change in FVCpp from Historical Placebo Data …………………………18

**Figure E4.** Adjusted Mean Change from Baseline in FVC Over Time ………………………………………………………19

**Figure E5.** Adjusted Mean Change from Baseline in FVCpp Over Time ………………………………………………….20

**Figure E6.** Time to IPF Progression ………………………………………………………………………………………………………..21

**4. Supplementary Tables**

**Table E1:** Operating Characteristics from Primary Analysis with Borrowing from

Historical Placebo …………………………………………………………………………………………………………………………………22

**Table of Contents (continued) Page**

**Table E2.** Representativeness of Study Participants ………………………………………………………………………………23

**Table E3.** Summary of Change from Baseline in FVC Over 26 Weeks …………………………………………………….24

**Table E4.** Summary of Change from Baseline in FVCpp Over 26 Weeks …………………………………………………25

**Table E5.** Preferred Term Grouping Categories ……………………………………………………………………………………..26

**Table E6.** Treatment-Emergent Adverse Events by Maximum Severity ………………………………………………….27

**Table E7.** Adverse Events of Special Interest ………………………………………………………………………………………….28

**Table E8.** Study Drug Related Treatment-Emergent Serious Adverse Events ………………………………………….29

**Table E9.** On-Treatment Death ………………………………………………………………………………………………………………30

**Table E10.** Treatment-Emergent Adverse Events Leading to Study Drug Discontinuation ………………………31

**5. References for Online Data Supplement** ………………….………………………………………………………………………34

1. **Members of the ELEVATE IPF Investigator Authorship Group**

Martin Maillo, M.D.; Good Air Institute, Santa Fe, Argentina

Miguel Bergna, M.D.; CEMER Medical Center for Respiratory Diseases, Buenos Aires, Argentina

Ramon A. Rojas, M.D.; Research Institute of Respiratory Diseases, Tucuman, Argentina

Luis Wehbe, M.D.; Emphysema Foundation, Mar del Plata, Buenos Aires, Argentina

Alexis Cazaux, M.D.; IMER Respiratory Medicine Institute, Cordoba, Argentina

Alejandro Chirino, M.D.; Breathe Comprehensive Clinical Health, Godoy Cruz, Argentina

Pedro Carlos Elias, M.D.; INSARES - Respiratory Health Institute, Mendoza, Argentina

Luciana Molinari, M.D.; Central Clinic S.A., Villa Regina, Argentina

Alicia Molina, M.D.; Dharma Medical Center, Mendoza, Argentina

Cristian Fazio, M.D.; Vistalba Health Center, Mendoza, Argentina

Victoria Kohn, M.D.; Aprillus Asistency and Research Center, Buenos Aires, Argentina

German Arce, M.D.; Medical Institute of Clinical Studies Foundation, Rosario, Santa Fe, Argentina

Ernesto Raso, M.D.; Centro Medico de Investigacion, San Rafael, Argentina

Matias Florenzano; M.D., Ph.D.; De los Andes Clinic, Santiago, Chile

Georgina Miranda, M.D.; CEC Center for Clinical Studies, Santiago, Chile

Juana Pavie Gallegos, M.D.; Integral Respiratory Center, Valparaiso, Chile

Absalon Rafael Silva Orellana, M.D.; Maule Clinic, Maule, Chile

Diana Cano, M.D.; Eastern Pulmonology Institute S.A., Bucaramanga, Colombia

Tatiana Valencia Castano, M.D.; Cardiomet CEQUIN Foundation, Armenia, Quindio, Colombia

David Tchkonia, M.D.; LTD Aversi Clinic, Tbilisi, Georgia

Kakha Vacharadze, M.D., Ph.D.; LTD The First Medical Center, Tbilisi, Georgia

Vakhtang Katsarava, M.D., Ph.D.; Tbilisi State Medical University and Ingorokva High Medical Technology University Clinic LLC, Tbilisi, Georgia

Nani Gonjilashvili, M.D., Ph.D.; Emergency Cardiology Center by Academician G. Chapidze LLC, Tbilisi, Georgia

Lali Kupreishvili, M.D.; National Center for Tuberculosis and Lung Diseases JSC, Tbilisi, Georgia

Katerina Antoniou, M.D., Ph.D.; University General Hospital of Heraklion, Heraklion, Greece

Aikaterini Manika, M.D., Ph.D.; General Hospital of Thessaloniki "G. Papanikolaou", Thessaloniki, Greece

Argyrios Tzouvelekis, M.D., Ph.D.; University General Hospital of Patras, Patras, Greece

Ioannis Tomos, M.D., Ph.D.; 5^th^ Pulmonary Medicine Department, SOTIRIA Chest Diseases Hospital of Athens, Athens, Greece

Efrosyni Manali, M.D.; University General Hospital "Attikon", Athens, Greece

Jaydip Deb, M.D.; Nil Ratan Sircar Medical College and Hospital, Kolkata, India

Sandeep Katiyar, M.D.; Apollo Spectra Hospital, Kanpur, India

Anjali R Nath, M.D.; Sparsh Super Specialty Hospital, Bangalore, India

Hafiz Deshmukh, M.D.; Mgm Medical College and Hospital, Aurangabad, Aurangabad, India

Tejas Kakkad, M.D.; Rhythm Heart Institute Vadodara, Vadodara, India

Rajesh Swarnakar, M.D.; Getwell Hospital and Research Institute, Nagpur, India

Gururaj Udachankar, M.D.; BHS Lakeview Hospital, Belagavi, India

Asish Deshmukh, M.D.; Oriion Citicare Super Specialty Hospital, Aurangabad, India

Syazatul Syakirin Sirol Aflah, M.D.; Institute of Respiratory Medicine, Kuala Lumpur, Malaysia

Yong-Kek Pang, M.D.; University Malaya Medical Centre, Department of Medicine, Kuala Lumpur, Malaysia

Noorul Afidza Muhammad, M.D.; Hospital Serdang, Clinical Research Center, Kajang, Malaysia

Megat Razeem Abdul Razak, M.D.; Hospital Tengku Ampuan Afzan, Kuantan, Malaysia

Irfhan Ali Hyder Ali, M.D.; Hospital Pulau Pinang, George Town, Malaysia

Chan Tha A Hing, M.D.; Hospital Sultanah Bahiyah, Bandar Alor Setar, Malaysia

Andrea Colli, M.D.; Oaxaca Site Management Organization S.C, Oaxaca, Mexico

Juan Francisco Moreno, M.D.; Dr. Jose Eleuterio Gonzalez Monterrey University Hospital, Monterrey, Mexico

Francisco Sanchez Llamas, M.D.; Occident Medical Unit, Zapopan, Mexico

Rodolfo Posadas Valay, M.D.; Integral Health Medical Unit, San Nicolas de los Garza, Mexico

Carlos Herrera Garcia, M.D.; CALPULAB, Tequixtlale, Mexico

Joel Santiaguel, M.D.; Philippine General Hospital, Manila, Philippines

Mae Campomanes, M.D.; St. Luke's Medical Center - Global City, Bonifacio Global City, Philippines

Pamela Joy Dionisio, M.D.; Lung Center of the Philippines, Quezon City, Philippines

Cristian Oancea, M.D.; "Dr. Victor Babes” Clinical Hospital of Infectious Diseases and Pneumophtisiology, Timisoara, Romania

Lavinia Davidescu, M.D.; S.C. Lavinia Davidescu Clinic SRL, Oradea, Romania

Cristian Cojocaru, M.D.; S.C. Netconsult SRL, Iasi, Romania

Riaz Suleman Dawood, M.D.; Ahmed Kathrada Lenmed Private Hospital, Johannesburg, South Africa

Ismail Kalla, M.D.; WITS Clinical Research, Johannesburg, South Africa

Larry Mey, M.D.; Ryexo Clinical Research, Pretoria, South Africa

Michael Van Der Linden, M.D.; Durban Lung Centre, Durban, South Africa

Paul Graham Williams, M.D.; Netcare Milpark Hospital, Parktown, South Africa

Jin Woo Song, M.D., Ph.D.; Asan Medical Center, University of Ulsan College of Medicine,

Seoul, South Korea

Yong Hyun Kim, M.D.; The Catholic University of Korea Bucheon St. Mary's Hospital, Bucheon-si, South Korea

Hyoung Kyu Yoon, M.D.; The Catholic University of Korea, Yeouido St. Mary's Hospital, Seoul, South Korea

Eun Kyung Kim, M.D.; CHA Bundang Medical Center, CHA University, Gyeonggi-do, South Korea

Hye Sook Choi, M.D.; Kyung Hee University Hospital, Seoul, South Korea

Sun Hyo Park, M.D.; Keimyung University - Dongsan Medical Center, Daegu, South Korea

Hongseok Yoo, M.D.; Samsung Medical Center, Seoul, South Korea

Won-Il Choi, M.D., Ph.D.; Hanyang University - Myongji Hospital, Goyang-si, South Korea

Yongchul Lee, M.D.; Chonbuk National University Hospital, Jeonju-si, South Korea

Yangjin Jegal, M.D.; Ulsan University Hospital, Ulsan, South Korea

Sung Hwan Jeong, M.D.; Gachon University Gil Medical Center, Incheon, South Korea

Hong-Joon Shin, M.D.; Chonnam National University Hospital, Gwangju, South Korea

DongWon Park, M.D.; Hanyang University Seoul Hospital, Seoul, South Korea

Jae Ha Lee, M.D.; InJe University Haeundae Paik Hospital, Busan, South Korea

Joo Hun Park, M.D., Ph.D.; Ajou University Hospital, Suwon, South Korea

Kittima Bangpattanasiri, M.D.; Central Chest Institute of Thailand, Nonthaburi, Thailand

Pailin Ratanawatkul, M.D.; Khon Kaen University, Khon Kaen, Thailand

Krittika Teerapuncharoen, M.D.; Siriraj Hospital, Bangkok, Thailand

Thomas P Jensen, M.S.; Berry Consultants, LLC, Austin, Texas, United States

Barbara Wendelberger, Ph.D.; Berry Consultants, LLC, Austin, Texas, United States

Farah Khandwala, M.S.; Berry Consultants, LLC, Austin, Texas, United States

Anna McGlothlin, Ph.D.; Berry Consultants, LLC, Austin, Texas, United States

Aaron Milstone, M.D.; Clinical Trials Center of Middle Tennessee, Franklin, Tennessee, United States

Abhishek Singla, M.D.; University of Cincinnati, Cincinnati, Ohio, United States

Juan Fernandez, M.D.; Harmony Medical Research Institute, Inc, Hialeah, Florida, United States

Mark Hamblin, M.D.; University of Kansas Medical Center, Kansas City, Kansas, United States

Tejaswini Kulkarni, M.D.; University of Alabama at Birmingham, Lung Health Center, Birmingham, Alabama, United States

Rafael Lupercio, M.D.; Paradigm Clinical Research Centers, Inc., Redding, California, United States

Benjamin Bregman, M.D.; Southeastern Research Center, Winston-Salem, North Carolina, United States

Richard Parisi, M.D.; TPMG Clinical Research – Williamsburg, Williamsburg, Virginia, United States

Yolanda Mageto, M.D.; Baylor Research Institute d/b/a Baylor Scott & White Research Institute, Dallas, Texas, United States

Damien Patel, M.D.; Indiana University Methodist Hospital, IU Health Enterprise Clinical Research Operations, Indianapolis, Indiana, United States

Toby Maher, M.D., Ph.D.; Keck School of Medicine, University of Southern California, Los Angeles, CA, United States

William Stringer, M.D.; The Lundquist Institute for Biomedical Innovation at Harbor-UCLA Medical Center, Torrance, California, United States

Ather Siddiqi, M.D.; Renovatio Clinical - The Woodlands Research Center, Woodlands, Texas, United States

Shilpa Johri, M.D.; Pulmonary Associates of Richmond, Richmond, Virginia, United States

Gerard Criner, M.D.; Temple University Hospital, Philadelphia, Pennsylvania, United States

Bruce Rankin, D.O.; Accel Research Sites Network, DeLand, Florida, United States

Ramana Puppala, M.D.; Clinical Research Investments, LLC, Decatur, Georgia, United States

Amy Case, M.D.; Piedmont Healthcare, Inc., Atlanta, Georgia, United States

Ryan Klein, M.D.; NewportNativeMD, Inc., Newport Beach, California, United States

Murali Ramaswamy, M.D.; Pulmonix, LLC, Greensboro, North Carolina, United States

Lisa Lancaster, M.D.; Vanderbilt University Medical Center, Nashville, Tennessee, United States

Todd Astor, M.D.; Science 37, Los Angeles, California, United States

Ameer Rasheed, M.D.; University of Connecticut Health Center, Farmington, Connecticut, United States

**Clinical Advisory Committee Members**

Marlies Wijsenbeek-Lourens, Rotterdam, The Netherlands; Kevin Flaherty, Ann Arbor, Michigan, USA; Paul Noble. Los Angeles, California, USA, Toby Maher, Los Angeles, California, USA, Williamson Bradford, Wilson, WY, USA; Vincent Cottin, Lyon, France

**Independent Data Monitoring Committee members**

Robert A. Wise, Baltimore, Maryland, USA; Ulrich Costabel, Essen, Germany; Shailendra S. Menjoge, Danbury, Connecticut, USA; Robert J. Kaner, New York, New York, USA; Lewis J. Smith, Chicago, Illinois, USA

**2. Supplementary Methods**

**Key Exclusion Criteria**

- Significant clinical worsening (as per investigator discretion) of IPF between Screening and Baseline Visits
- Current or prior treatment with pirfenidone or greater than six months treatment with nintedanib (current treatment with nintedanib excluded)
- Creatinine clearance <30 mL/min calculated by Cockcroft–Gault formula or chronic liver disease (Child-Pugh B or C hepatic impairment)
- Significant pulmonary hypertension or cardiovascular disease
- Primary obstructive airway physiology (prebronchodilator forced expiratory volume in the first second [FEV1]/FVC <0.7 at Visit 1)
- Known explanation for interstitial lung disease, including but not limited to radiation, sarcoidosis, hypersensitivity pneumonitis, bronchiolitis obliterans organizing pneumonia, human immunodeficiency virus (HIV), viral hepatitis, and cancer
- Diagnosis of any connective tissue disease, including but not limited to scleroderma/systemic sclerosis, Sjogren’s disease, mixed connective tissue diseases, polymyositis/dermatomyositis, systemic lupus erythematosus, and rheumatoid arthritis
- Use of prohibited drugs within 2 weeks prior to Visit 2/baseline or planned during the duration of the study
- Use of smoked (burned) tobacco products or vaping/e-cigarettes

**Further Endpoints**

**Secondary Efficacy Endpoint:**

- Time to hospitalization due to respiratory cause (as determined by the investigator) or all-cause mortality through 26 weeks

**Secondary Tolerability Endpoints:**

- Incidence of dose modifications (dose reductions and interruptions)
- Time to first dose modification (reduction or interruption)
- Duration of dose modifications (reductions and interruptions)
- Number of days on full assigned dose
- Incidence and duration of adverse events of special interest (AESIs)
- Time to treatment discontinuation due to an adverse event (AE)

**Exploratory Endpoints:**

- Time to hospitalization due to respiratory cause (as determined by the investigator) through 26 weeks
- Time to all-cause mortality through 26 weeks
- Change from baseline to Week 26 in King's Brief Interstitial Lung Disease Questionnaire (K‑BILD) total score
- Change from baseline to Week 26 in St. George’s Respiratory Questionnaire – IPF Version (SGRQ-I)
- Change from baseline to Week 26 in EuroQol 5-Dimensional Quality of Life Questionnaire (EQ-5D)
- Change in serum biomarkers from baseline to Week 26
- Number and duration of respiratory hospitalizations or pulmonary exacerbations (as determined by the investigator) through 26 weeks
- Rate of hospitalization due to respiratory cause (as determined by the investigator) through 26 weeks
- Proportion of patients with FVC change from baseline ≥0 at Week 26 (i.e., improvement or no decline in FVC)
- Pharmacokinetic endpoints

**Clinical trial sources and modeling used for dynamic borrowing in the placebo arm for Bayesian analyses**

**Historical external data for primary endpoint**

The primary efficacy analysis borrowed historical external placebo data from several IPF studies including the Phase 2 TOMORROW^1^ trial, Phase 3 INPULSIS^2^ trials, and Phase 3 ASCEND^3^ trial. These historical studies were selected because the baseline characteristics and background therapy profiles are very similar to the trial population enrolled to ELEVATE. Reported point estimates of placebo group change in FVC volume (mL) from baseline to week 52 from each trial were used to create prior point estimates of the annual rate of change in FVC volume. Estimates for each trial are reported and plotted in Figure S2, including an overall estimate from a generic inverse variance meta-analysis using a common effect model.

**Bayesian approach for primary endpoint**

The primary efficacy analysis was performed using a Bayesian linear mixed effects model. The response variable was the absolute FVC over time, including baseline. The model included fixed effects for treatment, time in weeks (as a continuous covariate), and treatment by time interaction, as well as subject-level random effects for the intercept and slope. The primary efficacy analysis of the ELEVATE study assessed the superiority of deupirfenidone to placebo as measured by the difference in rate of decline ($\theta$) in FVC between the combined deupirfenidone and placebo arms. The primary analysis would be declared successful if the posterior probability Pr($\theta$ > 0 | Data) > 0.9. The precise posterior probability that the difference exceeds 0 with 95% credible intervals is provided.

**Historical external data for key secondary endpoint**

The key secondary efficacy analysis borrowed historical external placebo subject data from the same IPF studies including the Phase 2 TOMORROW^1^ trial, Phase 3 INPULSIS^2^ trials, and Phase 3 ASCEND^3^ trial. Point estimates of the annual rate of change in FVCpp were constructed from reported point estimates of placebo population change in FVCpp from baseline to week 52 in each trial. Estimates for each trial are reported and plotted in Figure S3, including an overall estimate from a generic inverse variance meta-analysis using a common effect model.

**Bayesian approach for key secondary endpoint**

The key secondary endpoint analysis was performed using a similar Bayesian linear mixed effects model. The response variable was the absolute FVCpp over time, including baseline. The fixed effects included treatment, time in weeks (as a continuous covariate), and treatment by time interaction, as well as the random effects for the intercept and slope for each subject. The posterior probability threshold of 0.9 was chosen to be consistent with the hypothesis testing of the primary endpoint. The precise posterior probability that the difference exceeds 0 with 95% credible intervals is provided.

**Sample Size Determination**

The study was planned to randomize 1:1:1:1 ratio, a total of approximately N=240 participants, with 60 participants planned per arm. The primary efficacy evaluation was performed in the pooled deupirfenidone dose arms versus the placebo arm (120 vs 60 participants). The sample size was based upon a comparison of pooled deupirfenidone dose arms vs placebo and was sufficient to achieve 92.3% power to detect a true treatment effect using the primary Bayesian approach.

The primary outcome for this analysis was the rate of decline in FVC. The primary efficacy analysis was performed using a Bayesian linear mixed effects model with fixed effects for treatment arm, weeks (as a continuous variable), and an interaction term for weeks by treatment arm and random effects for intercepts and slopes.

The operating characteristics, including statistical power, of this trial were evaluated through virtual trial simulation. A normally distributed random slope had means ranging from –3.5 to –5.5 mL/week for placebo subjects. A range of standard deviations of the simulated random slopes and residual errors were explored for each treatment effect scenario. Table S1 lists the operating characteristics for a progression rate (random slope mean) of –4.5 mL/week for the placebo group, treatment effect sizes 0, 1.5, and 2.5 mL/week (i.e., progression rates of –4.5, –3.0, and –2.0 mL/week for the pooled treatment group), and standard deviation combinations of 4.7 mL/week and 43.3 mL, 6.7 mL/week and 62.0 mL, and 8.8 mL/week and 80.6 mL for the random slopes and residual standard deviations, respectively. For a treatment effect size of 2.5 mL/week and standard deviation combination 6.7 mL/week and 62.0 mL, the statistical power of the design is 92.3%; the one-sided type I error for a null effect in the same scenario is 6.1%. The posterior probability threshold 90% was chosen as successful criteria. The boundary of 90% is based on the simulations and balancing type I error rate for the further development of deupirfenidone. Refer to the Supplemental Appendix Statistical Analysis Plan for additional analysis operation characteristics.

**Data source**

Data represent the original December 4, 2024 database lock. On December 2, 2025, PureTech finalized an Errata that was judged to not substantially affect interpretation of the efficacy data. Briefly, the protocol defined the baseline for analysis as the most recent eligible spirometry results prior to administration of study drug. The vendor responsible for spirometry overread was asked to deliver data based on military time and did not account for the time change that occurs every spring and fall (DST versus STD) when programming this conversion. Five participants that originally were thought to have spirometry performed prior to first dose were discovered to actually have had spirometry performed within one hour after dosing once the military time conversion formula was corrected (one participant each on placebo, deupirfenidone 550 mg TID, and deupirfenidone 825 mg TID and two participants on pirfenidone). A sensitivity analysis substituted the last eligible spirometry values prior to administration of study drug (i.e., during screening) for these five participants. For the primary efficacy endpoint, FVC, the posterior probability demonstrated a very minor numeric difference of 98.4% compared to the 98.5% previously reported.

**3. Supplementary Figures**

**Figure E1. Study Design**

**
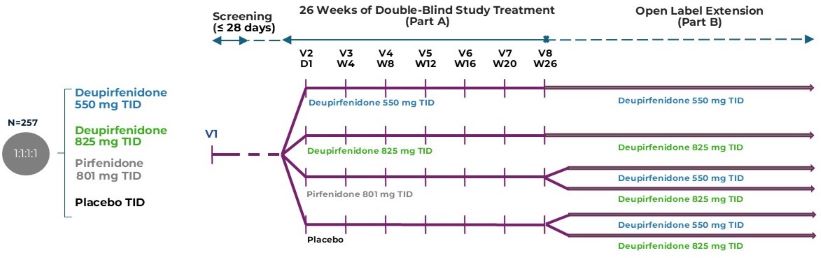
**

V = Visits; include a +/- 7-day window. TID = three times daily. Patients who completed Part A and chose not to continue with the Open Label Extension (OLE) had a final visit 28 days after completion of Part A. Patients who chose to enroll in OLE were assigned treatment allocations at entry into OLE and re-titrated to full assigned dose to maintain Part A study blind.

**Figure E2: Point Estimates of Annual Rate of Change in FVC Volume (mL/week) from Historical Placebo Data**

FVC = Forced viral capacity. Plots demonstrate 95% confidence intervals, respective standard error, and total reported placebo arm sample sizes.

**Figure E3: Estimates of Annual Rate of Change in FVCpp from Historical Placebo Data**


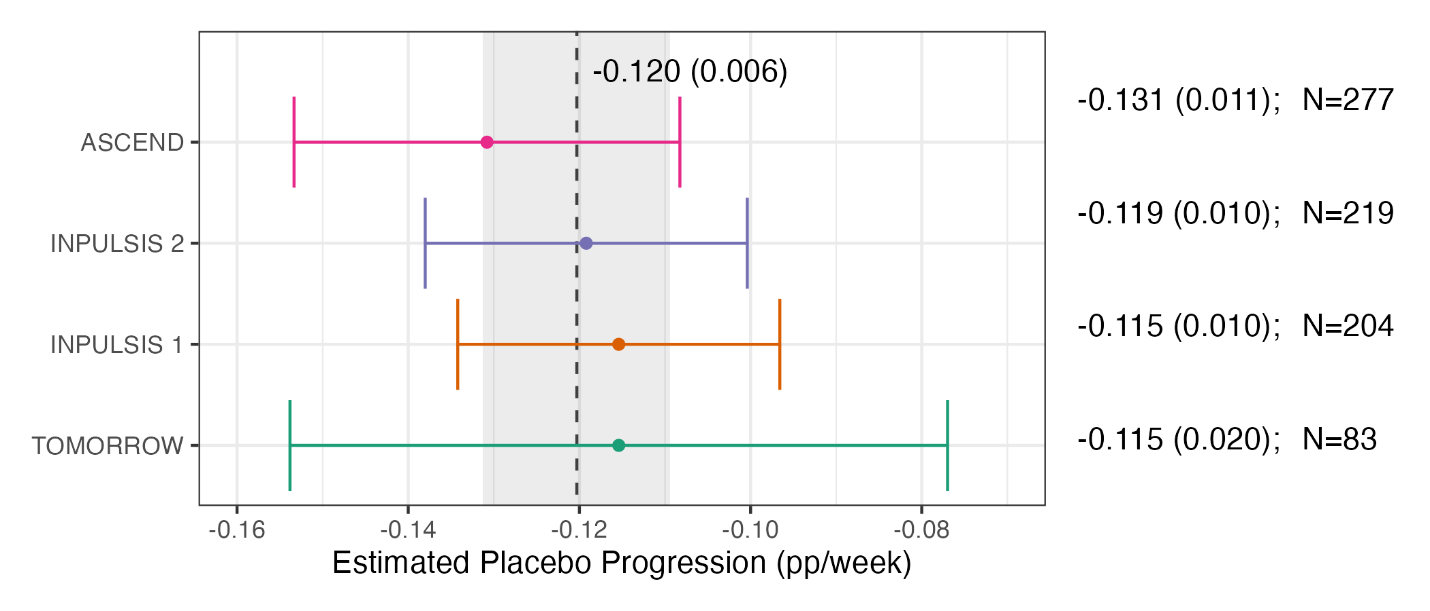


FVCpp = Forced vital capacity as a percent of predicted value. Plots demonstrate 95% confidence intervals, respective standard error, and total reported placebo arm sample sizes.

**Figure E4. Adjusted Mean (SE) Change from Baseline in FVC Over Time by Frequentist Approach**


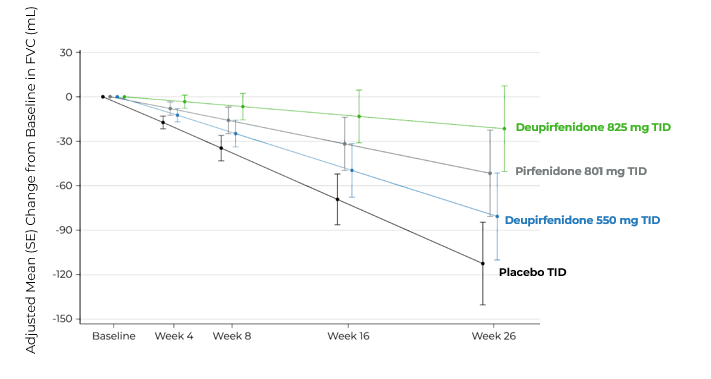


FVC = forced vital capacity; SE = standard error; TID = three times daily. Adjusted mean (SE) by Frequentist approach is estimated based on a random coefficient regression model with absolute FVC over time, including baseline, as a response, and fixed effects for treatment (placebo, pirfenidone, deupirfenidone 550 mg or deupirfenidone 825 mg), visit (week), and treatment by visit interaction, as well as participant-level random effects for the intercept and slope.

**Figure E5. Adjusted Mean (SE) Change from Baseline in FVCpp Over Time by Frequentist Approach**


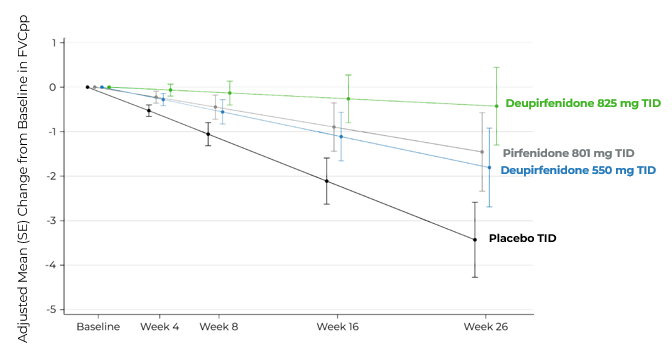
FVCpp = forced vital capacity percent predicted; SE = standard error; TID = three times daily. Adjusted mean (SE) by Frequentist approach is estimated based on a random coefficient regression model with FVCpp over time, including baseline, as a response, and fixed effects for treatment (placebo, pirfenidone, deupirfenidone 550 mg or deupirfenidone 825 mg), visit (week), and treatment by visit interaction, as well as participant-level random effects for the intercept and slope.

**Figure E6. Time to IPF Progression**

**
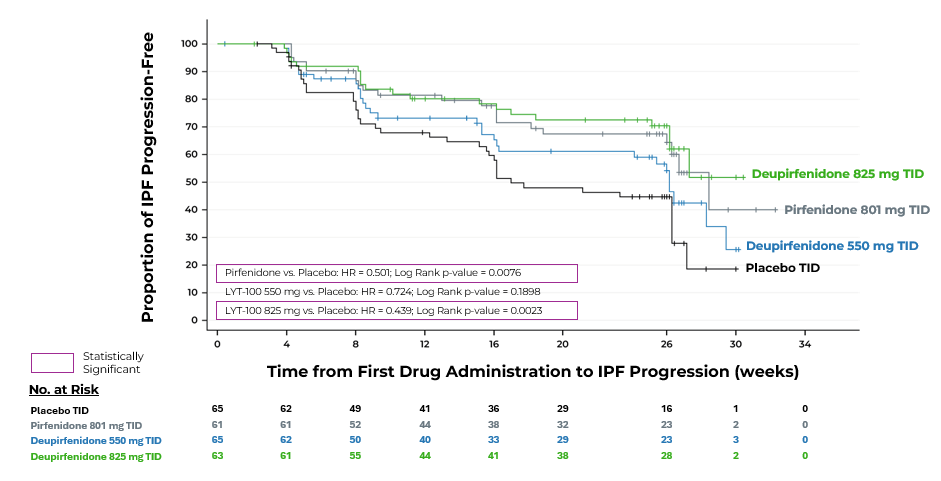
**

FVCpp = forced vital capacity percent of predicted value; IPF = idiopathic pulmonary fibrosis; TID = three times daily. IPF progression was defined as a decline in FVCpp of 5% or greater, or death. The median time to IPF progression for placebo was 17.0 weeks (95% CI, 15.1, 26.3), for pirfenidone 801 mg it was 28.4 weeks (95% CI, 26.0, not evaluable), for deupirfenidone 550 mg it was 26.1 weeks (95% CI, 16.1, 29.4). Deupirfenidone 825 mg delayed progression such that a median time was not evaluable (95% CI 26.14, not evaluable).

**4. Supplementary Tables**

**Table E1: Operating Characteristics from Primary Analysis with Borrowing from Historical Placebo**

| **Simulation Assumptions** | | | **Power** |
| --- | --- | --- | --- |
| **Placebo progression rate (mL/week)** | **Standard deviation: Random slope (mL/week) / Residual error (mL)** | **Treatment effect:**  **Rate effect deupirfenidone vs placebo**  **(mL/week)** | **Primary analysis:**  **dynamic borrowing** |
| **-4.5** | **4.7/43.3** | **0** | **0.062** |
|  |  | **1.5** | **0.810** |
|  |  | **2.5** | **0.991** |
|  | **6.7/62.0** | **0** | **0.061** |
|  |  | **1.5** | **0.593** |
|  |  | **2.5** | **0.923** |
|  | **8.8/80.6** | **0** | **0.061** |
|  |  | **1.5** | **0.441** |
|  |  | **2.5** | **0.780** |

**Table E2. Representativeness of Study Participants**

| **Category** |  |
| --- | --- |
| **Disease, problem, or condition under investigation** | Idiopathic pulmonary fibrosis (IPF) |
| **Special considerations related to:** |  |
| **Sex and gender** | Although some epidemiology studies suggest similar prevalence of IPF for men and women, clinical trials tend to enroll a higher percentage of men^4,5^. This may have to do with barriers to diagnosis or access to care. |
| **Age** | IPF primarily affects older adults; the majority are aged >65 years. The incidence of IPF increases with age.^5, 6^ |
| **Race and ethnic group** | Most racial and ethnic groups appear to have similar prevalence of IPF, with the exception of the Black/African American population potentially having lower prevalence^7^. It is unclear if the underrepresentation of this group in epidemiological studies is due to a true difference in prevalence or disparities in diagnosis. |
| **Geography** | While there is variability in the reported worldwide incidence and prevalence of IPF due to varying study methodologies, IPF remains a rare disease across geographies.^6^ |
| **Other considerations** | Globally, the prevalence of IPF is increasing, which may be attributed to aging populations, increased disease awareness, and/or improved diagnostic tools.^6^ |
| **Overall representativeness of this trial** | The sex and gender of the participants in this trial are representative of the broader IPF population. Participants were predominantly male with a mean age of 70.9. The geographies represented in this study were the United States (21%), Central/South America (27.2%), Europe and South Africa (19.8%), and Asia (31.9%). The racial distribution of the participants largely reflected the global nature of this study: 63% White or Caucasian, 33.5% Asian, 1.6% Black or African American, 1.9% Other, and 26.1% Hispanic or Latino. While the proportion of Black or African American patients included is representative of the proportion seen in large cohort studies, it is still unclear whether this is due to a true difference in prevalence or disparities in diagnosis.^7^ |

**Table E3. Summary of Change from Baseline in FVC Over 26 Weeks by Frequentist Approach**

| **Endpoint** | **Placebo TID (N=65)** | **Pirfenidone 801 mg TID (N=61)** | **Deupirfenidone 550 mg TID  (N=65)** | **Deupirfenidone 825 mg TID (N=63)** |
| --- | --- | --- | --- | --- |
| **Change from Baseline in FVC (mL)** | | | | |
| Adjusted Mean (SE) | -112.5 (27.84) | -51.6 (29.13) | -80.7 (29.32) | -21.5 (28.86) |
| 95% CI | (-167.2, -57.8) | (-108.8, 5.6) | (-138.3, -23.1) | (-78.2, 35.1) |
| **Comparison versus placebo** | | | | |
| Adjusted Mean Difference (SE) |  | 60.9 (40.29) | 31.8 (40.43) | 91.0 (40.10) |
| 95% CI |  | (-18.3, 140.0) | (-47.6, 111.2) | (12.2, 169.7) |
| P-Value |  | 0.1313 | 0.4322 | 0.0237 |

CI = confidence interval; FVC = forced vital capacity; SE = standard error; TID = three times daily. Baseline is defined as the last available measurement performed before the first study drug administration in Part A. Adjusted mean is estimated based on a random coefficient regression model with absolute FVC over time, including baseline, as a response, and fixed effects for treatment, visit (week), and treatment by visit interaction, as well as participant-level random effects for the intercept and slope.

**Table E4. Summary of Change from Baseline in FVCpp Over 26 Weeks by Frequentist Approach**

| **Endpoint** | **Placebo TID (N=65)** | **Pirfenidone 801 mg TID (N=61)** | **Deupirfenidone 550 mg TID  (N=65)** | **Deupirfenidone 825 mg TID (N=63)** |
| --- | --- | --- | --- | --- |
| **Change from Baseline in FVCpp** | | | | |
| Adjusted Mean (SE) | -3.43 (0.842) | -1.46 (0.881) | -1.81 (0.886) | -0.43 (0.872) |
| 95% CI | (-5.08, -1.77) | (-3.19, 0.28) | (-3.55, -0.07) | (-2.14, 1.29) |
| **Comparison versus placebo** | | | | |
| Adjusted Mean Difference (SE) |  | 1.97 (1.219) | 1.62 (1.222) | 3.00 (1.212) |
| 95% CI |  | (-0.42, 4.37) | (-0.78, 4.02) | (0.62, 5.38) |
| P-Value |  | 0.1060 | 0.1846 | 0.0135 |

CI = confidence interval; FVCpp = forced vital capacity percent of predicted value; SE = standard error; TID = three times daily. Adjusted mean is estimated based on a random coefficient regression model with absolute FVC over time, including baseline, as a response, and fixed effects for treatment, visit (week), and treatment by visit interaction, as well as participant-level random effects for the intercept and slope.

**Table E5. Preferred Term Grouping Categories**

| **Combined Term** | **Dyspepsia** | **Abdominal pain** | **Decreased appetite** | **Rash*** | **Upper Respiratory Infections** |
| --- | --- | --- | --- | --- | --- |
| Specific preferred terms that were aggregated into a single combined term | Dyspepsia | Abdominal pain | Decreased appetite | Rash | Upper respiratory tract infection |
|  | Gastroesophageal reflux disease | Abdominal discomfort | Food intolerance | Dermatitis† | Acute sinusitis |
|  | Gastritis | Abdominal pain lower | Hypophagia | Rash Maculo-papular | Bronchitis |
|  | Hyperchlorhydria | Abdominal pain upper |  | Rash erythematous | Sinusitis |
|  | Gastritis erosive | Abdominal fullness |  | Erythema | Tonsillitis |
|  |  | Abdominal tenderness |  |  | Tracheobronchitis |
|  |  | Epigastric pain lower |  |  | Tracheitis |
|  |  | Epigastric pain NOS |  |  | Pharyngitis |
|  |  | Epigastric pain of unknown origin |  |  | Nasopharyngitis |
|  |  | Epigastric discomfort |  |  |  |
|  |  | Abdominal distension‡ |  |  |  |

*Did not include photosensitivity reaction

†Did not include contact or allergic dermatitis

‡ “Bloating” was termed under abdominal distention

**Table E6. Number of Patients with Treatment-Emergent Adverse Events by Maximum Severity**

| **Category** | **Placebo TID (N=65) n (%)** | **Pirfenidone 801 mg TID (N=63) n (%)** | **Deupirfenidone 550 mg TID (N=65) n (%)** | **Deupirfenidone 825 mg TID (N=64) n (%)** | **Overall (N=257) n (%)** |
| --- | --- | --- | --- | --- | --- |
| Number of Patients with TEAEs | 48 (73.8) | 53 (84.1) | 47 (72.3) | 55 (85.9) | 203 (79.0) |
| **TEAEs by Maximum Severity** |  |  |  |  |  |
| mCTCAE Grade 1 | 18 (27.7) | 17 (27.0) | 12 (18.5) | 20 (31.3) | 67 (26.1) |
| mCTCAE Grade 2 | 21 (32.3) | 26 (41.3) | 22 (33.8) | 27 (42.2) | 96 (37.4) |
| mCTCAE Grade 3 | 5 (7.7) | 5 (7.9) | 11 (16.9) | 7 (10.9) | 28 (10.9) |
| mCTCAE Grade 4 | 2 (3.1) | 0 | 1 (1.5) | 0 | 3 (1.2) |
| mCTCAE Grade 5 | 2 (3.1) | 5 (7.9) | 1 (1.5) | 1 (1.6) | 9 (3.5) |

TEAE = treatment-emergent adverse event; mCTCAE = Modified Terminology Criteria for Adverse Events; TID = three times daily.

**Table E7. Adverse Events of Special Interest**

| **AESI Category***  **Preferred Term** | **Placebo TID (N = 65) n (%)** | **Pirfenidone 801 mg TID (N=63) n (%)** | **Deupirfenidone 550 mg TID (N=65) n (%)** | **Deupirfenidone 825 mg TID (N=64) n (%)** |
| --- | --- | --- | --- | --- |
| Participants with at Least 1 AESI | 1 (1.5) | 5 (7.9) | 2 (3.1) | 4 (6.3) |
| Anorexia, Decreased Appetite†, Fatigue | 0 | 3 (4.8) | 0 | 1 (1.6) |
| Fatigue | 0 | 2 (3.2) | 0 | 1 (1.6) |
| Decreased appetite | 0 | 2 (3.2) | 0 | 0 |
| Diarrhea, Nausea, Vomiting | 0 | 3 (4.8) | 0 | 1 (1.6) |
| Nausea | 0 | 1 (1.6) | 0 | 1 (1.6) |
| Diarrhea | 0 | 1 (1.6) | 0 | 0 |
| Vomiting | 0 | 1 (1.6) | 0 | 0 |
| Hepatic Laboratory Abnormalities | 1 (1.5) | 0 | 1 (1.5) | 2 (3.1) |
| Alanine aminotransferase increased | 0 | 0 | 1 (1.5) | 0 |
| Hepatic enzyme increased | 0 | 0 | 0 | 1 (1.6) |
| Liver function test increased | 1 (1.5) | 0 | 0 | 0 |
| Transaminases increased | 0 | 0 | 0 | 1 (1.6) |
| Photosensitivity Reaction and Rash† | 0 | 0 | 1 (1.5) | 0 |
| Photosensitivity reaction | 0 | 0 | 1 (1.5) | 0 |

AESI = adverse event of special interest; PT = preferred term; TID = three times daily

* The following events were considered AESIs if the event had a severity of Grade 3 or higher: anorexia, decreased appetite, fatigue; diarrhea, nausea, vomiting; increase in aspartate aminotransferase and/or alanine aminotransferase levels; photosensitivity reaction and rash. Participants who experienced multiple events within an AESI category or PT were counted once for each category and once for each PT.

†Decreased appetite and rash were grouped from multiple PTs; refer to Table SX. Preferred Term Grouping Categories

**Table E8. Study Drug Related Treatment-Emergent Serious Adverse Events**

| **System Organ Class  Preferred Term*** | **Placebo TID (N=65) n (%)** | **Pirfenidone 801 mg TID (N=63) n (%)** | **Deupirfenidone 550 mg TID (N=65) n (%)** | **Deupirfenidone 825 mg TID (N=64) n (%)** | **Overall (N=257) n (%)** |
| --- | --- | --- | --- | --- | --- |
| Participants with at Least 1 Study Drug-Related Treatment-Emergent SAE† | 2 (3.1) | 1 (1.6) | 0 | 1 (1.6) | 4 (1.6) |
| Gastrointestinal disorders | 0 | 1 (1.6) | 0 | 1 (1.6) | 2 (0.8) |
| Nausea | 0 | 0 | 0 | 1 (1.6) | 1 (0.4) |
| Vomiting | 0 | 1 (1.6) | 0 | 0 | 1 (0.4) |
| Hepatobiliary disorders | 1 (1.5) | 0 | 0 | 0 | 1 (0.4) |
| Bile duct stone | 1 (1.5) | 0 | 0 | 0 | 1 (0.4) |
| Cholangitis | 1 (1.5) | 0 | 0 | 0 | 1 (0.4) |
| Infections and infestations | 1 (1.5) | 0 | 0 | 0 | 1 (0.4) |
| Gastroenteritis | 1 (1.5) | 0 | 0 | 0 | 1 (0.4) |
| Investigations | 0 | 1 (1.6) | 0 | 0 | 1 (0.4) |
| Oxygen saturation decreased | 0 | 1 (1.6) | 0 | 0 | 1 (0.4) |

SAE = serious adverse event; TID = three times daily.

*Participants who experienced multiple events within a system organ class (SOC) or preferred term (PT) were counted once for each SOC and once for each PT at the strongest relationship reported.

† “Study drug related” were SAEs considered by the investigator to be related to study medication.

**Table E9. Details for Patients with On-Treatment Death**

| **Region** | **Age (decade)** | **Sex** | **Years from IPF diagnosis** | **FVCpp Baseline** | **Treatment arm** | **Days on study drug** | **Days from randomization to death** | **Cause of death** | **Related to study drug per PI** |
| --- | --- | --- | --- | --- | --- | --- | --- | --- | --- |
| Latin America | 50-60 | M | 0.4 | 66 | Placebo | 94 | 109 | Respiratory failure | No |
| Asia | 80-90 | M | 0.1 | 61.7 | Placebo | 136 | 136 | Suspected cardiac failure | No |
| North America | 80-90 | M | 4.0 | 41.57 | Pirfenidone 801 mg TID* | 28 | 65 | Acute on chronic respiratory failure | No |
| Latin America | 70-80 | M | 0.6 | 73.72 | Pirfenidone 801 mg TID | 119 | 132 | Multiorgan dysfunction syndrome | No |
| Latin America | 50-60 | F | 1.1 | 57.79 | Pirfenidone 801 mg TID | 143 | 163 | Community acquired bacterial pneumonia | No |
| Asia | 70-80 | M | 0.3 | Not available† | Pirfenidone 801 mg TID | 3 | 5 | Cardiorespiratory arrest | No |
| Asia | 60-70 | M | 0.1 | 50.14 | Pirfenidone 801 mg TID | 99 | 107 | Hospital acquired pneumonia | No |
| Asia | 60-70 | M | 0.2 | 69.75 | Deupirfenidone 550 mg TID | 61 | 70 | Community acquired pneumonia | No |
| Europe | 70-80 | M | 2.5 | 71.68 | Deupirfenidone 825 mg TID | 60 | 78 | Respiratory failure due to influenza | No |

*TID = three times daily

†No available spirometry that met quality standards

**Table E10. Treatment-Emergent Adverse Events Leading to Study Drug Discontinuation**

| **System Organ Classification  Preferred Term*** | **Placebo TID (N=65) n (%)** | **Pirfenidone 801 mg TID (N=63) n (%)** | **Deupirfenidone 550 mg TID (N=65) n (%)** | **Deupirfenidone 825 mg TID (N=64) n (%)** | **Overall (N=257) n (%)** |
| --- | --- | --- | --- | --- | --- |
| Participants with at Least 1 TEAE Leading to Study Medication Discontinuation | 8 (12.3) | 11 (17.5) | 16 (24.6) | 12 (18.8) | 47 (18.3) |
| Gastrointestinal disorders | 1 (1.5) | 5 (7.9) | 6 (9.2) | 4 (6.3) | 16 (6.2) |
| Nausea | 1 (1.5) | 3 (4.8) | 4 (6.2) | 2 (3.1) | 10 (3.9) |
| Diarrhea | 0 | 1 (1.6) | 2 (3.1) | 1 (1.6) | 4 (1.6) |
| Dyspepsia | 0 | 0 | 2 (3.1) | 1 (1.6) | 3 (1.2) |
| Abdominal pain | 0 | 1 (1.6) | 0 | 0 | 1 (0.4) |
| Vomiting | 0 | 0 | 1 (1.5) | 0 | 1 (0.4) |
| General disorders and administration site conditions | 0 | 5 (7.9) | 4 (6.2) | 1 (1.6) | 10 (3.9) |
| Fatigue | 0 | 3 (4.8) | 2 (3.1) | 1 (1.6) | 6 (2.3) |
| Asthenia | 0 | 0 | 2 (3.1) | 0 | 2 (0.8) |
| Chest discomfort | 0 | 0 | 0 | 1 (1.6) | 1 (0.4) |
| Chills | 0 | 1 (1.6) | 0 | 0 | 1 (0.4) |
| Multiple organ dysfunction syndrome | 0 | 1 (1.6) | 0 | 0 | 1 (0.4) |
| Pyrexia | 0 | 1 (1.6) | 0 | 0 | 1 (0.4) |
| Respiratory, thoracic and mediastinal disorders | 3 (4.6) | 2 (3.2) | 2 (3.1) | 2 (3.1) | 9 (3.5) |
| Idiopathic pulmonary fibrosis | 2 (3.1) | 0 | 2 (3.1) | 1 (1.6) | 5 (1.9) |
| Respiratory failure | 1 (1.5) | 0 | 0 | 1 (1.6) | 2 (0.8) |
| Dyspnea | 0 | 1 (1.6) | 0 | 0 | 1 (0.4) |
| Sneezing | 0 | 1 (1.6) | 0 | 0 | 1 (0.4) |
| Metabolism and nutrition disorders | 0 | 1 (1.6) | 4 (6.2) | 1 (1.6) | 6 (2.3) |
| Decreased appetite | 0 | 1 (1.6) | 4 (6.2) | 1 (1.6) | 6 (2.3) |
| Nervous system disorders | 0 | 2 (3.2) | 2 (3.1) | 2 (3.1) | 6 (2.3) |
| Headache | 0 | 2 (3.2) | 1 (1.5) | 0 | 3 (1.2) |
| Dizziness | 0 | 0 | 1 (1.5) | 1 (1.6) | 2 (0.8) |
| Cognitive disorder | 0 | 0 | 0 | 1 (1.6) | 1 (0.4) |
| Dysgeusia | 0 | 0 | 1 (1.5) | 0 | 1 (0.4) |
| Somnolence | 0 | 0 | 0 | 1 (1.6) | 1 (0.4) |
| Skin and subcutaneous tissue disorders | 0 | 2 (3.2) | 2 (3.1) | 2 (3.1) | 6 (2.3) |
| Rash | 0 | 1 (1.6) | 0 | 1 (1.6) | 2 (0.8) |
| Dermatitis allergic | 0 | 0 | 0 | 1 (1.6) | 1 (0.4) |
| Erythema multiforme | 0 | 1 (1.6) | 0 | 0 | 1 (0.4) |
| Photosensitivity reaction | 0 | 0 | 1 (1.5) | 0 | 1 (0.4) |
| Urticaria | 0 | 0 | 1 (1.5) | 0 | 1 (0.4) |
| Infections and infestations | 1 (1.5) | 2 (3.2) | 1 (1.5) | 1 (1.6) | 5 (1.9) |
| Pneumonia | 0 | 2 (3.2) | 1 (1.5) | 1 (1.6) | 4 (1.6) |
| Gastroenteritis | 1 (1.5) | 0 | 0 | 0 | 1 (0.4) |
| Influenza | 0 | 0 | 0 | 1 (1.6) | 1 (0.4) |
| Cardiac disorders | 1 (1.5) | 1 (1.6) | 2 (3.1) | 0 | 4 (1.6) |
| Cardiac failure | 1 (1.5) | 0 | 0 | 0 | 1 (0.4) |
| Cardio-respiratory arrest | 0 | 1 (1.6) | 0 | 0 | 1 (0.4) |
| Palpitations | 0 | 0 | 1 (1.5) | 0 | 1 (0.4) |
| Ventricular tachycardia | 0 | 0 | 1 (1.5) | 0 | 1 (0.4) |
| Psychiatric disorders | 0 | 1 (1.6) | 1 (1.5) | 2 (3.1) | 4 (1.6) |
| Depression | 0 | 0 | 1 (1.5) | 1 (1.6) | 2 (0.8) |
| Insomnia | 0 | 0 | 0 | 1 (1.6) | 1 (0.4) |
| Poor quality sleep | 0 | 1 (1.6) | 0 | 0 | 1 (0.4) |
| Investigations | 0 | 0 | 1 (1.5) | 2 (3.1) | 3 (1.2) |
| Alanine aminotransferase increased | 0 | 0 | 1 (1.5) | 0 | 1 (0.4) |
| Aspartate aminotransferase increased | 0 | 0 | 1 (1.5) | 0 | 1 (0.4) |
| Hepatic enzyme increased | 0 | 0 | 0 | 1 (1.6) | 1 (0.4) |
| Transaminases increased | 0 | 0 | 0 | 1 (1.6) | 1 (0.4) |
| Musculoskeletal and connective tissue disorders | 0 | 1 (1.6) | 0 | 1 (1.6) | 2 (0.8) |
| Arthralgia | 0 | 0 | 0 | 1 (1.6) | 1 (0.4) |
| Myalgia | 0 | 0 | 0 | 1 (1.6) | 1 (0.4) |
| Myopathy | 0 | 1 (1.6) | 0 | 0 | 1 (0.4) |
| Hepatobiliary disorders | 1 (1.5) | 0 | 0 | 0 | 1 (0.4) |
| Bile duct stone | 1 (1.5) | 0 | 0 | 0 | 1 (0.4) |
| Immune system disorders | 1 (1.5) | 0 | 0 | 0 | 1 (0.4) |
| Anti-neutrophil cytoplasmic vasculitis | 1 (1.5) | 0 | 0 | 0 | 1 (0.4) |

PT = preferred term; SOC = system organ class; TEAE = treatment-emergent adverse event; TID = three times daily.

*Participants who experienced multiple events within a SOC or PT were counted once for each SOC and once for each PT at the strongest relationship reported.

**5. References for Online Data Supplement**

E1. Richeldi L, Costabel U, Selman M, et al. Efficacy of a tyrosine kinase inhibitor in idiopathic pulmonary fibrosis. N Engl J Med 2011; 365:1079-87.

E2. Richeldi L, du Bois RM, Raghu G, et al. Efficacy and safety of nintedanib in idiopathic pulmonary fibrosis. N Engl J Med 2014; 370:2071-82.

E3. King TE Jr, Bradford WZ, Castro-Bernardini S, et al. A phase 3 trial of pirfenidone in patients with idiopathic pulmonary fibrosis. N Engl J Med. 2014; 370(22):2083-92.

E4. Jalbert, A-C., Siafa L, Ramanakumar, AV. et al. Gender and racial equity in clinical research for idiopathic pulmonary fibrosis: a systematic review and meta-analysis. Eur Respir J 2022; 59:2102969.

E5. Dempsey TM, Payne S, Sangaralingham L, Yao X, Shah ND, Limper AH. Adoption of the antifibrotic medications pirfenidone and nintedanib for patients with idiopathic pulmonary fibrosis. Ann Am Thorac Soc 2021;18(7):1121-1128.

E6. Maher, T.M., Bendstrup, E., Dron, L. et al. Global incidence and prevalence of idiopathic pulmonary fibrosis. Respir Res 2021;22:197.

E7. Adegunsoye A, Freiheit E, White EN, et al. Evaluation of pulmonary fibrosis outcomes by race and ethnicity in US adults. JAMA Netw Open 2023;6(3):e232427.
